# Data on the saturation behaviour of the 63-90 µm quartz from the Carpathian Basin

**DOI:** 10.1016/j.dib.2024.110702

**Published:** 2024-07-03

**Authors:** Zoran M. Perić, Cathal Ryan, Helena Alexanderson, Slobodan B. Marković

**Affiliations:** a Department of Geology, Lund University, Sölvegatan 12, SE-223 62 Lund, Sweden; b Irish Climate Analysis and Research Units, Department of Geography, Maynooth University, Maynooth, Ireland; c LAPER – Laboratory for Environmental reconstruction, Faculty of Science and Mathematics, University of Novi Sad, Trg Dositeja Obradovića 3, 21000 Novi Sad, Serbia; d Serbian Academy of Sciences and Arts. Knez Mihajlova 35, 11000 Belgrade, Serbia; e University of Montenegro. Cetinjska 2, 81000 Podgorica, Montenegro

**Keywords:** OSL dating, Maximum laboratory dose, Upper dating limit, Danube Basin, Loess

## Abstract

This dataset offers valuable insights into the luminescence saturation behaviour of 63–90 µm quartz grains sourced from the Carpathian Basin, as examined under controlled laboratory conditions. Its significance lies not only in shedding light on the luminescence properties specific to this region but also in facilitating comparative analyses with quartz samples from other geographic areas. Moreover, the dataset contributes novel findings to the ongoing investigations concerning the upper dating limit of quartz grains, which holds implications for refining luminescence dating methodologies. Grounded in the framework of several previous studies which underscore the challenges associated with utilizing quartz from certain regions for precise dose measurements, the dataset addresses the crucial aspect of setting upper dose limits for accurate luminescence dating. Consequently, the study conducts a series of tests to assess the proximity of natural sensitivity-corrected luminescence signals to laboratory saturation levels, particularly focusing on quartz samples from the Kisiljevo loess-palaeosol sequence. The dataset includes data from OSL saturation experiments conducted on sample 23019, along with associated calculations encompassing all 19 collected samples. This comprehensive dataset serves as a valuable resource for researchers and practitioners engaged in luminescence dating studies, offering detailed insights into saturation behaviours and dose-response characteristics of quartz grains from the Carpathian Basin. Beyond its immediate research implications, the dataset holds significant potential for reuse in various contexts. Researchers exploring luminescence properties of geological materials, particularly quartz grains, can leverage this dataset to compare saturation behaviours across different regions, thus enriching our understanding of luminescence dating methodologies on a broader scale. Additionally, the dataset could inform future studies on refining dose limits and calibration protocols, ultimately enhancing the accuracy and reliability of luminescence dating techniques. In summary, this dataset not only advances our understanding of luminescence saturation behaviours in quartz grains from the Carpathian Basin but also fosters collaborative research efforts aimed at refining luminescence dating methodologies and addressing broader questions in geochronology and palaeoenvironmental studies.

Specifications Table

**Table:** 

Subject	Earth and Planetary Sciences
Specific subject area	Chronostratigraphy.
Type of data	Analysed data, Table, Figure
Data collection	The luminescence data was collected through direct measurements of 63–90 µm quartz, using a Risø TL/OSL reader model DA-20. The reader was equipped with blue LEDs (470 nm, ∼80 mW.cm^−2^) and infrared (IR) LEDs (870 nm, ∼135 mW.cm^−2^). Irradiation was conducted using a ^90^Sr/^90^Y beta source. Quartz OSL signals were detected through 7.5 mm of Schott U-340 (UV) glass filter. The analysis of the luminescence data, including curve fitting and error calculations, was performed using the Analyst software version 4.57. Equivalent doses were measured via the Single Aliquot Regenerative Dose (SAR) protocol [1,2]. The purity of quartz grains was determined through the IR depletion test [3]. A maximum of 10% deviation from unity was deemed as acceptable for the recycling and IR depletion tests. The aliquots that did not meet these requirements were rejected and not used for the *D_e_* calculations.
Data source location	All analysed data are acquired from loess sediments collected at the Kisiljevo loess-palaeosol sequence in north-eastern Serbia (44°44′0′' N and 21°25′0′' E). The data is stored in The Lund Luminescence Laboratory, Department of Geology, Lund University, Sölvegatan 12, SE-223 62 Lund, Sweden.
Data accessibility	Repository name: Mendeley Data Data identification number: 10.17632/yxc6zd9kgc.1 Direct URL to data: https://data.mendeley.com/datasets/yxc6zd9kgc/1
Related research article	Z. M. Perić, C. Ryan, H. Alexanderson, and S. B. Marković, “Revised OSL chronology of the Kisiljevo loess-palaeosol sequence: New insight into the dust flux in the eastern Carpathian Basin during MIS 3 - MIS1,” Quat. Int., Jun. 2024, doi: 10.1016/j.quaint.2024.06.006 [4]

## 1 Value of the Data

• This data set provides information about the luminescence saturation behavior of 63–90 µm quartz grains from the Carpathian Basin in laboratory conditions.
• The provided records can be used to compare the luminescence properties with quartz from other regions.
• The analyzed data adds new information on the ongoing investigations of the upper dating limit of quartz grains.

## 2 Background

Wintle and Murray [5] proposed that, for the fast component of the quartz OSL signal, as measured through the SAR protocol, the upper dose limit should be set at 2×*D0*, corresponding to 86% saturation of the laboratory dose-response curve. This agrees with the findings of Timar-Gabor and Wintle [6], Timar-Gabor et al [7]. and Perić et al [8]. for Romanian and Serbian loess respectively, where it was concluded that quartz from this region cannot be used for measuring a known given dose in the high dose range and that most measurements overestimated the expected doses. Here we performed three tests in order to estimate the closeness of the natural sensitivity-corrected luminescence signal to laboratory saturation for all quartz samples However, given that the quartz from the Kisiljevo loess- palaeosol sequence exhibited no proximity to the laboratory saturation level, we conducted two additional saturation tests to investigate the quartz characteristics in more detail. The data presented here is the summary of the performed measurements.

## 3 Data Description

The dataset presented here and in Perić et al [9]. contains the data from the performed OSL saturation experiments conducted on sample 23,019 and the associated calculations on saturation levels on all 19 collected samples. The excel file labeled G3097-de_sat [10] contains the measurement data and analysis from the saturation test, generated by the Analyst software, version 4.57 [11]. Column A, row 11 in the G3097-de_sat excel file shows the reader dose rate expressed in Gy. Column F shows the measured equivalent doses (ED) in seconds, while column G (De) presents the calculated equivalent doses for all measured aliquots in Gy. Column H shows the calculated mean equivalent doses for: the natural signal (rows 3–5); natural signal with an added dose of 511 Gy (rows 6–7); natural signal with an added dose of 1022 Gy (rows 8–9). Column I presents the calculated uncertainty of the mean doses in column H. The recycling ration is presented in column T (RR1), while the measured recuperation (recup1) is shown in column AD. The upper and lower integration limit of the luminescence signal (Sig1 and Sig2) used for the calculation of the equivalent doses are shown in columns AO and AP respectively. The upper and lower background limit (BG1 and BG2) subtracted from the net signal are presented in columns AQ and AR respectively.

Table 1. Shows the natural corrected luminescence signals for the Kisiljevo samples and maximum corrected luminescence signals recorded in the SAR-OSL protocol. The last column shows the ratio of the natural signal to the signal measured for a given dose of 11 680 Gy on sample 23,019, used as an indicator of the proximity of the signal to saturation.

**Table 1:** Summary of the saturation level calculation for all measured quartz samples from the Kisiljevo LPS.

Measurement	Sample ID	L_n_/T_n_ mean	L_x_/T_x_ 11,680 Gy	(L_n_/T_n_)/(L_x_/T_x_)
OSL	23,001	1.93±0.16	14.11±1.76	0.14±0.02
	23,002	6.86±0.24	14.11±1.76	0.49±0.06
	23,003	6.57±0.33	14.11±1.76	0.47±0.06
	23,004	5.75±0.15	14.11±1.76	0.41±0.05
	23,005	5.98±0.15	14.11±1.76	0.42±0.05
	23,006	5.72±0.15	14.11±1.76	0.41±0.05
	23,007	4.72±0.08	14.11±1.76	0.33±0.04
	23,008	4.60±0.11	14.11±1.76	0.33±0.04
	23,009	3.90±0.07	14.11±1.76	0.28±0.03
	23,010	5.62±0.22	14.11±1.76	0.40±0.05
	23,011	5.27±0.19	14.11±1.76	0.37±0.05
	23,012	4.62±0.09	14.11±1.76	0.33±0.04
	23,013	5.33±0.14	14.11±1.76	0.38±0.05
	23,014	4.81±0.15	14.11±1.76	0.34±0.04
	23,015	4.66±0.13	14.11±1.76	0.33±0.04
	23,016	4.28±0.09	14.11±1.76	0.30±0.04
	23,017	5.29±0.16	14.11±1.76	0.38±0.05
	23,018	4.72±0.14	14.11±1.76	0.33±0.04
	23,019	5.55±0.17	14.11±1.76	0.39±0.05

The data presented in Table 2 displays corrected luminescence signals for sample 23,019 from Kisiljevo for doses of 511 and 1024 Gy, given on top of the natural dose, alongside the highest corrected luminescence signals detected in the SAR-OSL procedure. The last column indicates the ratio of the natural signal to the signal detected at a dose of 11 680 Gy on sample 23,019, serving as an indicator of signal saturation proximity.

**Table 2:** Summary of the conducted saturation tests on sample 23,019.

Measurement	Sample ID	L_n_/T_n_ mean	L_x_/T_x_ 10,000 Gy	(L_n_/T_n_)/(L_x_/T_x_)
SAR-OSL	23,019 (511 Gy)	2.49±0.14	14.11±1.76	0.18±0.02
	23,019 (1024 Gy)	2.07±0.13	14.11±1.76	0.15±0.02

Table 3 shows the information on the ratios between the expected *D_e_*s (calculated by adding the given dose to the measured natural dose) and the measured doses obtained in the saturation test for quartz sample 23,019.

**Table 3:** Relationship between given to measured doses obtained in the saturation test.

Sample	Natural dose (Gy)	Given dose (Gy)	Expected *D_e_*	Measured *D_e_*	Ratio
23,019	82.1±8.5	511	593.1±8.5	525.9±16.3	0.89±0.03
	82.1±8.5	1022	1104.1±8.5	995.1±40.2	0.90±0.04

**Fig. 1:**
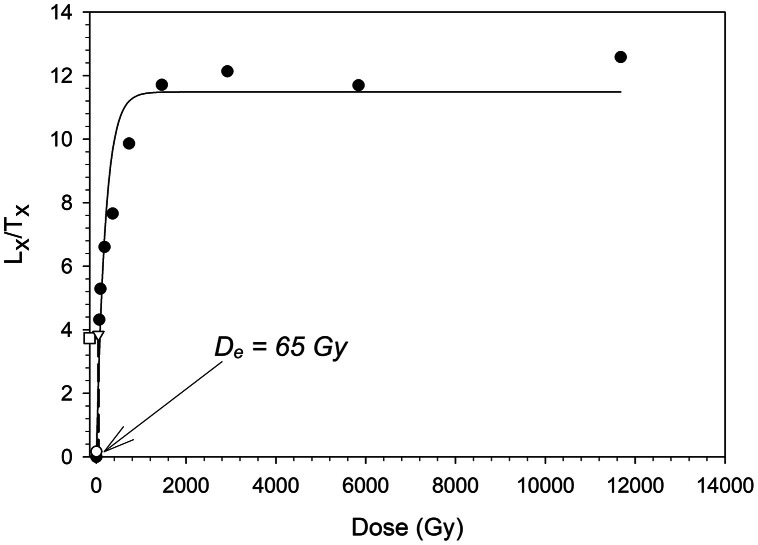
Representative dose response curve from one natural aliquot of sample 23,019.

In Fig. 1 we show a representative dose response curve from a single aliquot of sample 23,019. The data is derived from the saturation test performed on natural aliquots. The given doses were ∼46, 91, 183, 365, 730, 1460, 2920, 5840 and 11,680 Gy. The natural measured dose was 65.1±5.4 Gy. The dose-response curve was fitted by a single saturating exponential function. The *D_e_* value for this aliquot was determined by interpolating the sensitivity-corrected natural luminescence signal on the dose-response curve (indicated by the dashed line). The open downward triangle denotes the remeasured dose point (recycling ratio), the open circle represents the response to zero dose (recuperation), and the open square illustrates the sensitivity-corrected natural OSL signal.

**Fig. 2:**
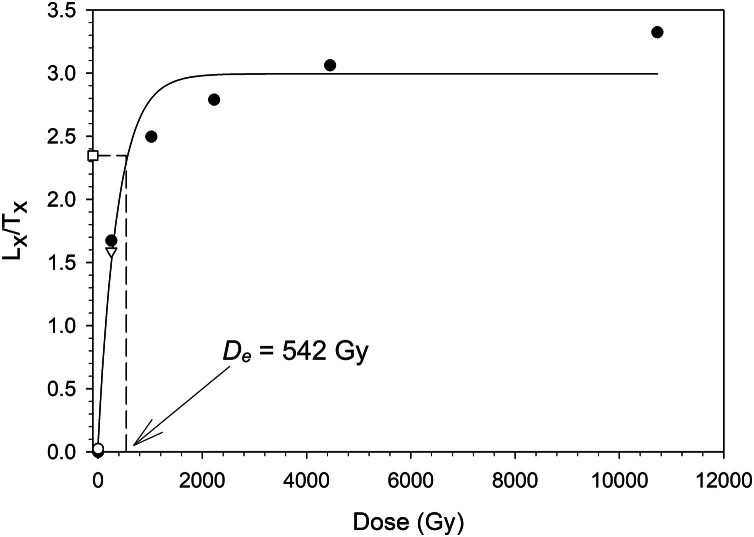
Dose response curve for one aliquot of sample 23,019 where a dose of 511 Gy was added to the natural dose.

The data presented in Fig. 2 results from the saturation test where a dose of 511 Gy was added on top of the natural dose. For the construction of the dose response curve, additional doses of ∼255, 1022, 2226, 4452 and 10,728 Gy were administered. The measured dose for this aliquot was 542.2±49.7 Gy. The sensitivity corrected natural signal is depicted as an open square and the dashed line shows the equivalent dose. The response to a zero dose is depicted as open circle. The recycling point is represented as an open inverted triangle.

**Fig. 3:**
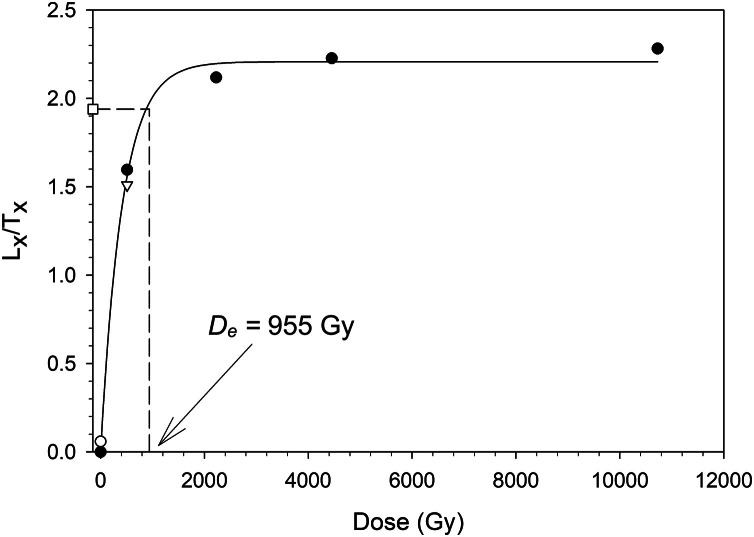
Dose response curve for one aliquot of sample 23,019 with an added dose of 1022 Gy on top of the natural dose,.

The data depicted in Fig. 3 is the results of the saturation test with a dose of 1022 Gy added on top of the natural dose. The dose response curve was constructed by inducing additional doses ranging from ∼255 to 10,728 Gy. The dose for this measurement was 955.0±121.2 Gy. The open square depicts the sensitivity corrected natural signal while the dashed line shows the equivalent dose. Recuperation (response to a zero dose) is represented by an open circle. The recycling point is depicted as an open inverted triangle.

## 4 Experimental Design, Materials and Methods

**Fig. 4:**
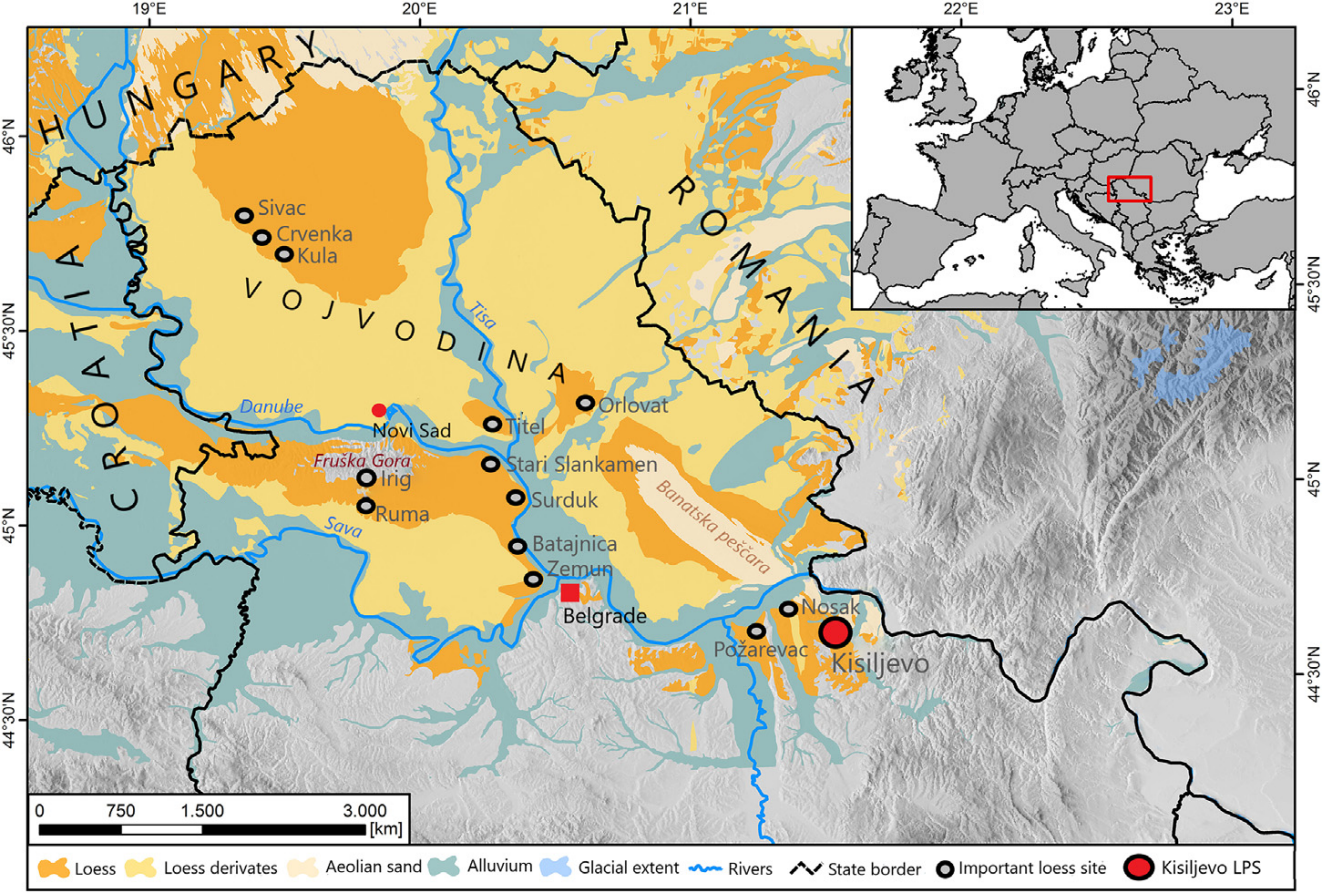
Map showing the geographical position of the sampling site (Kisiljevo LPS) and other important loess sites in the Carpathian Basin. The distribution of loess, loess derivates, aeolian sand and alluvium are presented according to Lehmkuhl et al. [12]. Modified from Perić et al. [13].

The samples for this study were obtained in November 2022 by driving stainless steel tubes into the freshly cleaned loess profile. A total of 19 samples were collected at a resolution of 50 cm, spanning depths from 10 cm to 900 cm (Fig. 4).

These samples were processed at the Lund luminescence laboratory, Lund University, Sweden, under conditions of low-intensity red light. The inner material of the cylinders was utilized for measuring equivalent dose (*D_e_*). For the *D_e_* measurements the 63–90 µm material was chosen for sample preparation. This sediment underwent treatment with 10% HCL (for 60 minutes) and 10% H_2_O_2_ (for 60 minutes) to remove carbonates and organic matter, respectively. To isolate the quartz fraction, the material was submerged in a sodium heteropolytungstate solution (2.62 g cm^−3^) for 24 hours. The >2.62 g cm^−3^ quartz fraction was then treated with a 40% HF solution for 90 minutes to eliminate any remaining feldspar contamination, followed by a 10% HCL treatment for 120 minutes to remove fluorides. After each treatment, the samples underwent three washes with deionized water. The quartz grains were then mounted as large 8 mm aliquots on stainless steel discs using silicone oil spray as an adhesive. All the measurements were performed using two Risø TL/OSL DA-20 readers equipped with blue LEDs (470 nm, ∼80 mW.cm^−2^) and infrared (IR) LEDs (870 nm, ∼135 mW.cm^−2^). The quartz OSL signals were collected through 7.5 mm of Schott U-340 (UV) glass filter. Irradiation was carried out using a 90^Sr^/90^Y^ beta source calibrated using the Risø calibration quartz, batch 123 [14]. The calibrated dose rates varied between 0.1861 and 0.1783 Gy for Reader 1 and 0.1516–0.1507 Gy for reader 2. For all measurements the Single Aliquot Regenerative Dose (SAR) protocol, was used [1,2]. Quartz grains were subjected to measurements with preheat and cut-heat temperatures set at 240 °C and 200 °C, respectively. Optical stimulation was administered for 40 seconds at 125 °C using blue LEDs. The pertinent luminescence signal for analysis was integrated over the initial 0.16 seconds of the decay curve, with an early background (interval of 0.32–0.48 seconds) subtracted from the net signal. Sensitivity changes were rectified by utilizing the OSL signal to a test dose (10% of the expected dose). Following each SAR cycle, a high-temperature bleach at 280 °C was carried out for 40 seconds. For the saturation experiment the OSL signal was fitted using a single saturating exponential function. The rejection criteria were based on the intrinsic tests performance: recycling ration, IR depletion and recuperation. A tolerance of a 10% deviation from unity was applied for recycling and IR depletion tests, while for the recuperation signal, 5% of the natural signal was considered to be within acceptable values. The raw luminescence data were analyzed using the Risø Analyst software, version 4.57 [11]

To assess the expected doses for the saturation test and determine the average maximum corrected luminescence signal we induced doses ranging from 46 to 11 680 Gy (9 dose points) on a natural aliquot of sample 23,019. The maximum corrected luminescence signal was established by constructing a laboratory dose response curve up to 11 680 Gy (average value of three measured aliquots).

The saturation test, as outlined by Timar-Gabor and Wintle [6], involved applying large doses of 511 and 1022 Gy on top of the natural dose on sample 23,019 (with 3 aliquots per dose). One aliquot per dose was discarded due to a depletion ratio exceeding 10% (outside uncertainty). Following this, dose-response curves were plotted up to approximately 11 000 Gy.

## Limitations

All the measurements were conducted on 63–90 µm quartz from a single loess-palaeosol sequence. This grain size was chosen because of its abundance and the experience from previous studies on OSL dating of loess from the Kisiljevo site [13]. The acquired data is therefore limited to one grain size and one site only and should not be used as a standard for general luminescence properties for all quartz from the Carpathian Basin.

## Ethics Statement

The authors have read and follow the ethical requirements for publication in Data in Brief and confirm that the current work does not involve human subjects, animal experiments, or any data collected from social media platforms.

## CRediT Author Statement

**Zoran M. Perić:** Conceptualization, Methodology, Validation, Formal analysis, Investigation, Data curation, Writing – original draft, Writing – review & editing, Visualization. **Cathal Ryan:** Sample collection, Review and editing. **Helena Alexanderson:** Formal analysis, Investigation, Review and editing. **Slobodan B. Marković:** Sample collection, Review and editing.

## Data Availability

Data on saturation measurements of the Kisiljevo quartz (Original data) (Mendeley Data)
